# A Case of Sloughing of the Uterus

**Published:** 1871-11

**Authors:** W. G. Drake

**Affiliations:** Atlanta, Ga.


THE GEORGIA

### MEDICAL COMPANION:

A MONTHLY ADVISER.

VOL. I. ATLANTA, GA., NOVEMBER, 1871. NO. 11

### PART I.

### Original Communications and Special Selections.

### A CASE OF SLOUGHING OF THE UTERUS.

BY W. G. DRAKE, M. D., OF ATLANTA, GA.

On the 3d of August, 1860, while practicing in Alabama, I was called to visit a negro woman in her first confinement; being absent at the time, Dr. B. visited the case, in the afternoon of that day, and its history, up to the time of my arrival on the 4th, as gathered from him, is about as follows:

He learned from the nurse, that the woman had been complaining since the 2d. Making the necessary examination, he found the os-uteri dilated and without any rigidity, but as all effort upon the part of the womb, at expulsion, had ceased, he commenced the administration, vini ergotæ, in drachm doses, at short intervals. Waiting sufficiently long for the specific effects of the drug to be produced, and none being developed, he concluded, as there were no alarming symptoms present, to retire for the night, with instructions to the nurse, that he be called if anything should occur requiring his attention. He was not disturbed, and found on visiting the woman, in the morning, that she had passed, comparatively, a comfortable night, with only slight contractions, at long intervals; at this time, however, being more frequent and of greater force, ergot was again administered, and acted well until the head had thoroughly descended and become impatced between the pelvic bones, when nature ceased her efforts, and the ergot lost its effects.

Waiting several hours, and everything remaining "in statu quo," I was again sent for with the request, that I bring obsterical instruments. Soon after arriving I instituted an examination and found firm impaction of the fetal head, with the corrugations of the scalp protruding beyond the labia. I proposed the immediate application of the forceps—but the Dr. suggested the propriety of waiting as he had a short while previous to my arrival, again resorted to the use of ergot.

An hour or two having elapsed and the uterus still remaining inert, an effort was made to apply the instruments, but we found it impossible to lock them until craniotomy was performed, after which, with the aid of the forceps, and without difficulty, quite a large and perfectly developed male fetus was delivered.

On the passage of the head there gushed forth an unusually large quantity of decomposed liquor amnii, of creamy consistency, the color of lemon, and an ordor very offensive, so much so indeed, that I found it impossible to remain in the room without vomiting.

There was no difficulty attending the removal of the placenta, nor was the post partum hemorrhage, more considerable than usual with primipora, though the uterus failed to contract kindly until ergot was given, and ice-water applied externally and over the uterine region.

The nervous system was very much depressed and reaction, though gradual, seemed to be perfect under the use of alcoholic stimuli. I remained with the patient that night, and left her next morning doing as well as could be expected, after a protracted labor, and, an instrumental delivery.

I visited the case five or six days consecutively, and no untoward symptoms occurring, she was discharged. There had not been up to that time, any indication of metritis or metro-peritonitis, mammary abscess, nor any of the other "ills" to which lying-in-women "are heir to"—the patient remaining cheerful, the apetite good, the bowels moving regularly, without calling into requisition the aid of the usual remedies on such occasions. In fact, I never attended a case that gave promise of a more favorable termination.

Thirteen days subsequent to my last visit, I was again sent for, and on my arrival, was informed by the nurse, that the woman's bladder "was out." On making an examination I found, instead of that viscus being prolapsed, I had a case of procidentia uteri, with the lips and vaginal cervix, &c., a state of sphacelus. I introduced a catheter to the fundus, through which passed, probably, a-half pint of limpid fluid, and not very offensive. Then grasping the protruding part between the thumb and index finger, I made gentle pressure, by degrees approaching the body of the organ until I reached a point of sensibility, and just below that point and above the surgical neck I excised the insensible portion.

Various astringents and antisceptic remedies were applied locally, hoping thereby, to arrest further decomposition and complete destruction of the organ, and as the functions of assimilation and digestion were somewhat impaired, the vegetable and ferruginous tonics were administered with apparent benefit. I noticed, however, on each visit, that the womb had gradually descended, and on one occasion, I cought hold of it and made gentle traction, and no pain being complained of, I continued to pull, when, to my utter astonishment, there came away the entire mass with its follapian appendages. From that time, the woman improved rapidly in general health, and it was but a short while, until she was competent to perform all her duties as house-servant.

About eighteen months subsequent to the coming away of the womb, she informed me that she came unwell regularly, or in other words, there was a periodical discharge of blood from the vagina, corresponding with the catamenial flow, which occurred regularly prior to the time of her conception, and, at times, the sexual orgasm was as intense, and the sensations peculiar to the act of coition were as *exquisitely acute* as before her confinement.

The case, I must confess, is to me an unique one, (never having seen a similar one recorded, and in conversation in regard to it, with medical men, have had them express their doubts as to its being truly the uterus, but, instead, a false membrane,) and I do not pretend to account for the above facts, except upon the supposition that both, or at least, one of the ovaries, remained intact. Having an opportunity in 1862, I made a digital examination and found that quite a change had taken place in the physical condition of the vagina, its walls, as well as the sphincter vaginæ, were very much contracted, affording considerable resistance to the introduction of the finger, and in a measure accounting for the cessation of the stillicidium urinæ, with which she was at first troubled. I saw her again in 1865. She informed me that she enjoyed perfect health, and suffered no inconvenience from being minus a womb. I still have the specimen in alcohol, and those who may be skeptical, can have an opportunity of examining it, by calling at my office.

